# Results of Medium Seventeen Years' Follow-Up after Laparoscopic Choledochotomy for Ductal Stones

**DOI:** 10.1155/2016/9506406

**Published:** 2016-01-03

**Authors:** Silvia Quaresima, Andrea Balla, Mario Guerrieri, Giovanni Lezoche, Roberto Campagnacci, Giancarlo D'Ambrosio, Emanuele Lezoche, Alessandro M. Paganini

**Affiliations:** ^1^Department of General Surgery, Surgical Specialties and Organ Transplantation “Paride Stefanini”, Sapienza University of Rome, 00185 Rome, Italy; ^2^Department of General Surgery, Polytechnical University of Marche, 60121 Ancona, Italy

## Abstract

*Introduction*. In a previously published article the authors reported the long-term follow-up results in 138 consecutive patients with gallstones and common bile duct (CBD) stones who underwent laparoscopic transverse choledochotomy (TC) with T-tube biliary drainage and laparoscopic cholecystectomy (LC). Aim of this study is to evaluate the results at up to 23 years of follow-up in the same series. *Methods*. One hundred twenty-one patients are the object of the present study. Patients were evaluated by clinical visit, blood assay, and abdominal ultrasound. Symptomatic patients underwent cholangio-MRI, followed by endoscopic retrograde cholangiopancreatography (ERCP) as required. *Results*. Out of 121 patients, 61 elderly patients died from unrelated causes. Fourteen patients were lost to follow-up. In the 46 remaining patients, ductal stone recurrence occurred in one case (2,1%) successfully managed by ERCP with endoscopic sphincterotomy. At a mean follow-up of 17.1 years no other patients showed signs of bile stasis and no patient showed any imaging evidence of CBD stricture at the site of choledochotomy. *Conclusions*. Laparoscopic transverse choledochotomy with routine T-tube biliary drainage during LC has proven to be safe and effective at up to 23 years of follow-up, with no evidence of CBD stricture when the procedure is performed with a correct technique.

## 1. Introduction

Laparoscopic cholecystectomy (LC) is the gold standard treatment for symptomatic cholecystolithiasis. The most appropriate management of concurrent common bile ductal stones (CBDS) in the elective setting, however, remains controversial. CBDS are present in approximately 10–15% of patients undergoing cholecystectomy for symptomatic gallstones [1, 2] and its incidence increases with advancing age [3]. After the introduction of LC, the diagnosis and management of CBDS have largely relied on preoperative detection and clearance by endoscopic retrograde cholangiopancreatography (ERCP) in patients with suspected CBDS based on clinical indicators (history, laboratory exams, and ultrasound). This is associated with between 20% and 60% of negative and therefore useless endoscopic procedures, due to the low predictive value of the clinical indicators of ductal stones [4]. When ductal stones are confirmed, ERCP fails to clear the ducts in 7–14% of cases, and it is the cause of added morbidity and mortality [5–7], including 9–12% long-term recurrence rate of ductal stones, which is a cause of concern particularly in younger patients [8]. The introduction of less invasive imaging techniques, such as magnetic resonance cholangiography (CMRI) [9] and endoscopic ultrasound (EUS) [10], eliminates the need for a purely diagnostic ERCP but they increase the diagnostic burden for the patient and increase costs.

The improved laparoscopic skill and the development of dedicated laparoscopic instrumentation have offered the opportunity to treat gallstones and CBDS laparoscopically during the same session. This option, which has been introduced since more than 20 years into routine surgical practice and has become routine in a few centers, has proven to be a safe and effective alternative to the sequential endolaparoscopic approach. The EAES clinical trial comparing LC and common bile duct exploration (LCBDE) versus ERCP with endoscopic sphincterotomy (ES) followed by LC in fit patients (American Society of Anesthesiologists (ASA) grades I and II) has proven the two approaches to be equally effective but with a shorter hospital stay after the single-stage approach [5]. However, this is still considered a highly demanding procedure requiring a prolonged learning curve, suturing skills, and dedicated instrumentation. The transcystic duct approach does not require the suture skills that are needed to perform a laparoscopic choledochotomy and are considered by some as preferable to a choledochotomy also in patients presenting with acute cholecystitis [11]. Other authors consider a laparoscopic choledochotomy to be better indicated [12, 13] in case of large or impacted ductal stones, a narrow cystic duct, or stones located in the hepatic duct, because in these cases the failure rate of the transcystic duct approach increases. The short- and long-term results of the single-stage laparoscopic approach are reported in the literature, showing the safety and efficacy of these techniques also in terms of stone recurrence, common bile duct stricture, and cholangitis rates [14–19]. In a previously published paper the authors reported the follow-up results at an average of 72.3 months (range 11–145 months) in a series of 138 unselected, consecutive patients who underwent laparoscopic transverse choledochotomy and ductal clearance during LC [12]. Aim of this study is to evaluate the longer-term results at an average of 17 years (range 12–23 years) of follow-up in the same case series.

## 2. Materials and Methods

**Figure 1:**
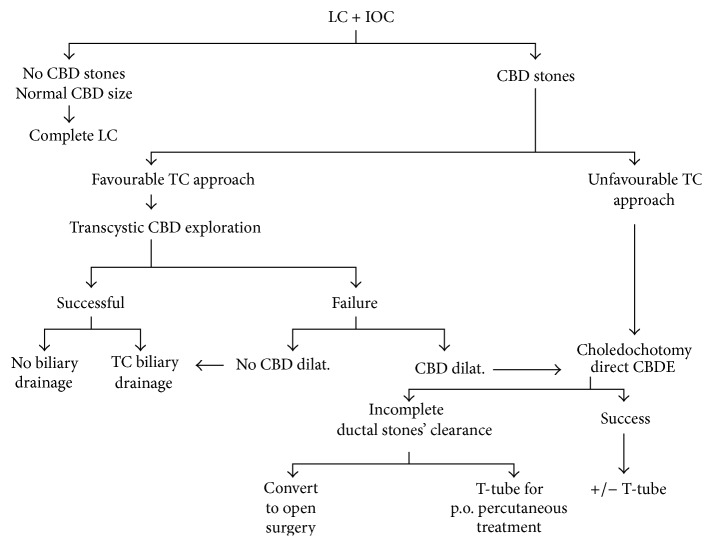
Treatment algorithm for patients undergoing laparoscopic cholecystectomy with intraoperative cholangiography. LC: laparoscopic cholecystectomy. IOC: intraoperative cholangiography.

The patients presenting gallbladder stones and secondary CBD stones were treated according to the authors' previously published algorithm and surgical technique (Figure 1) [20]. Study design is a retrospective analysis of prospective collected data and includes the 121 patients who were present at the end of the previous follow-up study and are the object of the present study [12]. All patients have at least a 12 years' duration of follow-up and they were evaluated by clinical visit, symptoms' questionnaires form completion, blood assay, and abdominal ultrasound (US). Symptomatic patients underwent MRI, with operative ERCP if treatment was required.

## 3. Results and Discussion

### 3.1. Results

Out of 121 cases, 61 patients who were elderly at the time of surgery had passed away for unrelated causes but were declared free from biliary symptoms from their relatives. Fourteen patients (11.5%) were lost to follow-up. The 46 remaining patients from the original series underwent the follow-up protocol (17 males and 29 females were examined; mean age was 76.4 years, range 45–92 years), with a medium follow-up of 17.1 years (range 12.6–22.7 years).

Specific symptoms of bile stasis occurred in one (2.1%) female patient presenting with episodes of cholangitis that occurred sixteen years after LC + LCBDE. Two more (4.3%) male patients reported dyspepsia. Biochemistry was negative in all patients except for the patient with cholangitis who showed increased levels of alkaline phosphatase, *γ*-glutamyl transpeptidase, and transaminases together with leukocytosis. One of the two patients reporting dyspepsia had moderately increased transaminases levels but no biochemical signs of bile stasis. US evaluation was performed in every patient (100%) while CMRI was obtained in symptomatic patients only. No stones or biliary sludge was observed at US in 45 patients (97.83%). In the only symptomatic patient no ductal stones were seen at US. This patient underwent CMRI that showed the presence of two stones measuring 1 and 0.8 cm, respectively, located in the distal common bile duct. ERCP with sphincterotomy was performed, with stones' removal. Neither signs of papillitis nor of ductal stricture at any level of the extrahepatic bile ducts were observed. In this case a mild increase in pancreatic enzymes occurred on the first day after ERCP-ES but subsequently resolved and the patient was discharged on postprocedural day III, after normalization of biochemical parameters. Follow-up data are summarized in Table 1.

### 3.2. Discussion

Aim of this study is to report the longer-term results with a medium follow-up of 17 years in a consecutive series of unselected patients who underwent laparoscopic choledochotomy with T-tube biliary drainage. Complete follow-up data, including physical examination, laboratory exams, and imaging data, were obtained in the 46 patients who were available at the time of the follow-up call. Sixty-one patients had passed away for other reasons since many of them were already older than 65 years of age at the time of surgery [21]. To the best of our knowledge, this is the only series reported to date with a minimum follow-up longer than 12 years, and ranging up to almost 23 years, after single-stage laparoscopic treatment of gallstones and CBD stones by laparoscopic choledochotomy with T-tube biliary drainage.

Single-stage laparoscopic treatment of gallstones and CBD stones has been adopted by few dedicated centers [5, 14, 15, 22–24] but it is slowly gaining favor [25]. When a two-stage approach is followed, ERCP even in large series has its own morbidity and mortality (5–9,8% and 0.3-2,3%, resp.) [5, 6, 26–29], which adds up to those of LC. The most frequently reported complications after ERCP-ES are acute postprocedural pancreatitis, bleeding from the papilla, and duodenal perforation, events that are almost never observed after the single-stage approach [5, 6]. Moreover, at long-term follow-up recurrent stones and cholangitis occur in 9–12% of cases after ERCP + ES, due to the subsequent modifications of the normal physiologic barrier and bactibilia [16]. Some studies have also reported an increased rate of difficult LC and higher conversion rates after ERCP, even if the previous endoscopic procedure had been only diagnostic [30–32]. As for the success rates, in the past several randomized multi-institutional trials have shown equivalence of single-stage versus the two-stage approach [5, 11, 33]. However, the overall operative times and the length of hospital stay were shorter and significantly in favor of the single-stage treatment [5]. According to these data, the two-stage treatment should be reserved to patients at high risk for laparoscopic surgery (ASA III-IV) and to emergency patients with cholangitis or pancreatitis [34, 35]. More recently, a meta-analysis [36] including 1410 patients and 15 randomized controlled trials reported statistically significant differences in terms of ductal clearance rate, operative time, hospital stay, and cost, again in favor of the one-stage treatment. A systematic review of 16 randomized trials [37] comparing one-stage surgical versus two-stage endosurgical management of CBDS reported equivalent short-term results, except for the retained stones' rate, hospital stay, and hospital charge rates, which were lower after the single-stage approach. Previous international guidelines [38] considered treatment of CBDS to be mandatory, even if asymptomatic. A more recent revision of the indications suggests a conservative attitude considering that in more than one-third of patients spontaneous stones passage occurred uneventfully [39, 40]. The 2006 guidelines of the European Society for Endoscopic Surgery (EAES) [41] justified the conservative approach, especially in elderly patients with asymptomatic stones.

In the authors' opinion, laparoscopic transcystic duct exploration should be the technique of choice because it is less invasive. Laparoscopic choledochotomy should be reserved to patients in whom transcystic duct exploration is not possible or when intraoperative cholangiography shows the presence of unfavorable conditions for a transcystic duct approach. The ideal indications for the transcystic duct approach have been clearly defined: (a) a dilated cystic duct, joining the CBD on its lateral side; (b) a limited number of ductal stones (<4); (c) small size of ductal stones located in the CBD [13, 16, 42]. On the other hand, in case of large or impacted CBD stones, hepatic duct stones, Mirizzi syndrome, previous Billroth II gastrectomy, or Roux-en-Y gastric bypass for morbid obesity, a laparoscopic choledochotomy is better indicated since it provides direct access to the biliary tracts, improving the ductal clearance rate [43].

Patients who undergo a choledochotomy are generally considered to be at greater risk of long-term morbidity, namely, stricture, also up to twenty years after surgery as reported after open surgery [44]. The main cause of ductal stricture after choledochotomy is the occurrence of mucosal ischemia during the surgical procedure, as it may occur after inappropriate use of electrocautery or when the surgeon performs an extensive longitudinal choledochotomy. Other causes of major bile duct stricture are recurrent episodes of cholangitis, direct trauma from stones, pancreatitis, and lymphadenopathy [45]. Strictures that may have been due to technical mistakes, such as the use of a T-tube drainage that is too large as compared to the diameter of the ductal lumen, are also reported [46]. In the present series, the laparoscopic choledochotomy was short and it was performed transversely in every case. Such a short and transverse incision is aimed at avoiding interruption of the transversely oriented arterioles that are recognized by the laparoscopic magnified vision on the anterior wall of the CBD. These arterioles originate from the two main arteries running at hours three and nine along the major bile duct [47]. Suture of a transverse choledochotomy eliminates the risk of lumen reduction that may instead occur after suture of a long longitudinal choledochotomy, when the CBD may in fact take an “hourglass” appearance after completion of the suture [48]. Moreover, the authors have always employed only small diameter (3-4 mm), Silcolatex (Teleflex Medical Europe Ltd., IDA Business Park, Athlone, Co. Westmeath, Ireland) T-tubes, which have been kept in place for at least four weeks to allow the formation of a mature sinus tract, so as to avoid the occurrence of bile peritonitis after biliary drainage removal. The fact that no bile duct stricture was observed in this series questions the theory that the presence of a T-tube by itself may be the cause of a stricture, as long as the choledochotomy is performed correctly and the chosen T-tube is also correct. Although the choice of a transverse choledochotomy is not supported by a randomized trial comparing the two techniques, which would require enrolment of too large a number of patients to reach statistical significance, still the absence of bile duct strictures observed in the present series and in the previously published one [12] confirms that the authors' hypotheses may be correct. Other authors reported absence of strictures following choledochotomy in studies with short-medium follow-up time (36–68 months) [19, 45, 49], but only one recently published randomized controlled trial comparing LCBDE versus the two-stage approach reports differences in biliary strictures' rate (LCBDE 2.06% versus two-stage 0%, not statistically significant) and recurrent CBD stones rate (LCBDE 2,06% versus ERCP + LC 9,47%; *p* = 0,037) following longitudinal choledochotomy at long-term follow-up (8–10 years) [50].

In the authors' series, a T-tube drainage was employed routinely after laparoscopic transverse choledochotomy because the aim was to evaluate the safety and efficacy of this (by that time) new procedure, particularly in terms of residual stones' rate. Later, the indications for T-tube placement were reviewed and now it is employed selectively only in case of intraoperative instrumental manipulation of the papilla, such as transpapillary passage of basket or choledochoscope. Several other complications related to the use of T-tube biliary drainage are reported in the literature and include fluid electrolyte imbalance, sepsis, premature dislodgement, bile leak, biliary peritonitis, prolonged biliary fistula, and late biliary stricture [51, 52]. Some authors report that T-tube placement* per se* does not prevent the occurrence of bile leaks, which has been reported when the drainage is* in situ* as well as after its removal [51, 52]. A Cochrane meta-analysis [53] compared the results of T-tube placement versus primary closure of the choledochotomy and concluded that primary closure was safer and more effective, reducing the morbidity rate and hospital stay as compared to T-tube placement. At the beginning of the present series, three patients had a biliary leak due to T-tube kinking which resolved spontaneously. With improved experience, no other case of biliary leak was observed. Presently, the authors support primary closure of the transverse choledochotomy by a running suture, and T-tube placement is reserved only for patients with difficult bile duct clearance requiring some type of papilla manipulation.

The main criticisms against laparoscopic single-stage treatment of gallstones and CBD stones are related to purported higher costs, the need for advanced laparoscopic biliary expertise, and the fact that the operative time for LC with LCBDE may not be foreseen in every case. The cost of LC with LCBDE has been reported by several authors to be lower than for the two-stage approach [54–56]. Concerning the need for highly dedicated training required to accomplish LCBDE, at least two studies have shown no statistically significant differences in terms of morbidity and mortality rates, as well as length of hospital stay comparing the results of expert versus nonexpert surgeons in LCBDE during LC, with only the operative time being significantly different between the two groups [57, 58]. The main criticism to the present study is the lacking of a control group; however the good results obtained, also at long-term follow-up, are not substantially different from those published by other authors [17, 50]. The low recurrent stones rate and the absence of stricture are due to the standardization of the protocol treatment, the long surgical learning curve, and a very low endoscopic sphincterotomy rate. As reported in the literature sphincterotomy is the cause of a higher recurrent stones rate at long-term follow-up (up to 9–12%), related to an iatrogenic dysfunction of an otherwise normally functioning sphincter of Oddi.

## 4. Conclusion

In conclusion, in the authors' opinion LCDBE during LC should be the treatment of choice in elective patients with gallstones and CBD stones in centers where adequate expertise and a dedicated instrumentation are available. This procedure should be performed by a laparoscopic transcystic duct approach whenever possible and by transverse choledochotomy in selected cases. Laparoscopic transverse choledochotomy during LC has proven to be safe and effective also at longer-term follow-up, with no evidence of common bile duct stricture and with a stones' recurrence rate that is much lower than that reported in the literature after endoscopic sphincterotomy. For good results to be achieved, the choledochotomy must be performed with a correct surgical technique aimed at preventing the occurrence of ischemia, with a T-tube of small diameter, which is left in place long enough for a mature sinus tract to develop, so as to avoid the occurrence of bile peritonitis after biliary drainage removal.

## Figures and Tables

**Table 1:** Results of medium 17 years' follow-up (12.6–22.7 years).

Patients, *n*	*121*

Unrelated death, *n* (%)	61 (50.4)
Lost at follow-up, *n* (%)	14 (11.5)
Available patients' data, *n* (%)	**46 (38.1)**

Symptomatic patients, *n* (%)	**3 (6.5)**
Cholangitis	1 (2.7)
Dyspepsia	2 (5.4)

Biochemical biliary stasis, *n* (%)	**1 (2.1)**

US evaluation, *n* (%)	**46 (100)**
Stones	0 (0)
Stricture	0 (0)

MRI, *n* (%)	**1 (2.1)**
Stones	1 (2.1)
Ductal dilatation	1 (2.1)

Ductal stones' recurrence, *n* (%)	**1 (2.1)**

ERCP, *n* (%)	**1 (2.1)**

CBD stricture, *n* (%)	**0 (0)**
